# Appendectomy as a Risk Factor for Bacteremic Biliary Tract Infection Caused by Antibiotic-Resistant Pathogens

**DOI:** 10.1155/2017/3276120

**Published:** 2017-05-15

**Authors:** Koki Kawanishi, Jun Kinoshita, Hiroko Abe, Tetsuhiro Kakimoto, Yuko Yasuda, Takeshi Hara, Jun Kato

**Affiliations:** ^1^Department of Gastroenterology, Wakayama Rosai Hospital, 93-1 Kinomoto, Wakayama City, Wakayama 640-8505, Japan; ^2^Second Department of Internal Medicine, Wakayama Medical University, 811 Kimiidera, Wakayama City, Wakayama 641-0012, Japan

## Abstract

**Background/Aims:**

Recent evidence has suggested that appendix plays a pivotal role in the development and preservation of intestinal immune system. The aim of this study is to examine whether prior appendectomy is associated with an increased risk for the development of antibiotic-resistant bacteria in bacteremia from biliary tract infection (BTI).

**Methods:**

Charts from 174 consecutive cases of bacteremia derived from BTI were retrospectively reviewed. Using multivariate analysis, independent risk factors for development of antibiotic-resistant bacteria were identified among the clinical parameters, including a history of appendectomy.

**Results:**

In total, 221 bacteria strains were identified from 174 BTI events. Of those, 42 antibiotic-resistant bacteria were identified in 34 patients. Multivariate analysis revealed that prior appendectomy (Odds ratio (OR), 3.02; 95% confidence interval (CI), 1.15–7.87; *p* = 0.026), antibiotic use within the preceding three months (OR, 3.06; 95% CI, 1.26–7.64; *p* = 0.013), and bilioenteric anastomosis or sphincterotomy (OR, 3.77; 95% CI, 1.51–9.66; *p* = 0.0046) were independent risk factors for antibiotic-resistant bacteria.

**Conclusions:**

Prior appendectomy was an independent risk factor for the development of antibiotic-resistant bacteria in bacteremia from BTI.

## 1. Introduction

Biliary tract infection (BTI) with bacteremia represents 8–20% of community-acquired bacteremia in the elderly population and it is the second most common cause of sepsis in those subjects, leading to 10–20% of mortality [1, 2].

Bacteremia in BTI is caused by a wide spectrum of pathogens. In recent years, the prevalence of antibiotic-resistant pathogens in BTIs has risen steadily [1, 2]. Infectious conditions caused by those pathogens have also been associated with treatment failure and higher mortality [1, 3]. Several risk factors for the development of antibiotic-resistant bacteria from BTI have been identified, including healthcare-associated infection, previous hospitalization, previous antibiotic use and bilioenteric anastomosis or sphincterotomy, indwelling biliary drainage, and malignancy [2, 4, 5]. Bacterial pathogens are not always isolated during the course of bacteremia and there is inevitable delay in the identification of susceptibility to antibiotics even in subjects with positive blood culture. Therefore, knowledge of intrinsic resistance patterns of common biliary pathogens is essential for empiric therapy.

Previously, the human appendix has been regarded as a rudimentary part of the intestine. In recent years, however, several studies have suggested its immunological importance for the development and preservation of the intestinal immune system [6]. In addition, the appendix has been shown to have an important interaction with intestinal flora and is believed to work as a “safe house” for commensal gut flora [7]. In this sense, the appendix potentially serves to reinoculate the intestine with normal flora when unbeneficial pathogens arise in the gut [7]. Moreover, it has been reported that prior appendectomy is associated with the recurrence and fulminant course of* Clostridium difficile* infection [8, 9] and recurrence of small intestinal bacterial overgrowth after antibiotic treatment [10]. In this regard, appendectomy may be correlated with antibiotic resistance in patients with severe bacterial infections.

The focus of the current study is to analyze whether prior appendectomy is associated with an increased risk of the development of antibiotic-resistant bacteria in bacteremic BTI.

## 2. Materials and Methods

### 2.1. Patients and Study Design

This retrospective study was conducted at the Wakayama Rosai Hospital in Wakayama, Japan. Charts from 174 consecutive cases of bacteremia derived from BTI treated in the hospital between June 2005 and May 2016 were retrospectively reviewed. Patients with the following criteria were excluded from the study: (i) positive culture for probable skin contaminants (i.e., coagulase-negative* Staphylococci*,* Corynebacterium*,* Propionibacterium*, and* Bacillus species*) and (ii) bacteremia due to a non-BTI.

Medical records were reviewed and the following information was collected: age, sex, severity of infection (assessed using Pitt bacteremia score), cause of infection, underlying diseases, medication, history of appendectomy, previous antibiotic use (within 90 days), presence of indwelling biliary device, previous hospitalization (within 30 days), and history of bilioenteric anastomosis or sphincterotomy. In addition, 30-day mortality of the patients was examined.

This study was approved by the institutional review board at the Wakayama Rosai Hospital and conforms to the provisions of the Declaration of Helsinki (as revised in Fortaleza, Brazil, October 2013).

### 2.2. Definitions

Cholecystitis was diagnosed on the basis of clinical presentation of fever, right upper quadrant pain, and findings of ultrasonography or computed tomography. Cholangitis was diagnosed based on the following criteria: (a) presence of fever with upper quadrant pain; (b) radiological (sonographic or computed tomographic) or endoscopic evidence of biliary tract obstruction due to stones or stricture from benign or malignant origin; and (c) laboratory findings of hyperbilirubinemia and an elevated serum alkaline phosphatase level.

The Pitt bacteremia score was used to assess the severities of bacteremic BTIs and was calculated as follows:

- Oral temperature: two points for a temperature of ≤35°C or ≥40°C or one point for a temperature between 35.1 and 36.0°C or between 39.0 and 39.9°C
- Hypotension: two points for an acute hypotensive event with decreases in systolic (>30 mmHg) and diastolic (>20 mmHg) blood pressures, the use of intravenous vasopressor agents, or systolic blood pressure of <90 mmHg
- Receiving mechanical ventilation: two points
- Suffering cardiac arrest: four points
- Mental status: being alert, zero points; being disoriented, one point; being stuporous, two points; and being comatose, four points [11]

Antibiotic-resistant bacteria were defined as extended-spectrum *β*-lactamase- (ESBL-) producing bacteria, ampicillin-resistant* Enterococci* and SPACE (*Serratia*,* Pseudomonas*,* Acinetobacter*,* Citrobacter*, and* Enterobacter*), in this study according to the previous reports [12–14].

### 2.3. Microbiological Investigation

Two sets of two 8–10 mL blood samples were taken from patients who presented with ≥38°C or who showed clinical signs or symptoms associated with bacteremia. Blood samples were processed in the Oxoid Signal Blood Culture System (Kanto Kagaku, Tokyo, Japan) between June 2005 and October 2011 and processed in an automated BacT/ALERT 3D System (SYSMEX bioMérieux, Tokyo, Japan) between November 2011 and May 2016. The incubation period of blood samples was five days. Positive blood samples were subcultured onto blood agar plates or Brucella blood agar plates. Identification of aerobic and anaerobic bacteria was performed by the WalkAway System (Beckman Coulter) and RapID ANA II (AMCO Inc., Tokyo, Japan), respectively.

### 2.4. Statistical Analysis

Differences between patients with and without antibiotic-resistant bacteria were determined using the chi-square test and Student's *t*-test as appropriate. Independent risk factors for the detection of antibiotic-resistant bacteria were identified using multivariate logistic regression analysis that included parameters with *p* < 0.1 in univariate analysis. *p* value < 0.05 was considered statistically significant and odds ratios (ORs) with 95% confidence intervals (CIs) were determined. Data were statistically analyzed using JMP version 9 software (SAS Institute, Cary, NC, USA) [15].

## 3. Results

### 3.1. Clinical Characteristics of Patients

The demographic and clinical features of analyzed patients with bacteremia are summarized in Table 1. A total of 174 (mean age, 70.2 ± 13.9 y; male, 106, 61%) patients with bacteremia due to BTI were identified. The mean value of the Pitt bacteremia score was 1.22 ± 1.21. Thirty-two (18%) patients had history of appendectomy. Antibiotic use within the preceding three months, indwelling biliary devices, hospitalization within the preceding 30 days, and bilioenteric anastomosis or sphincterotomy were observed in 57 (33%), 56 (32%), 51 (29%), and 42 (24%) patients, respectively. Fourteen patients (8%) died within 30 days of admission.

### 3.2. Microbiological Characteristics

The bacteria strains isolated from the blood cultures are listed in Table 2. In total, 221 bacteria strains were identified from 174 BTI events.* Klebsiella *species were the most common isolates (83 out of 221, 38%), followed by* Escherichia coli* (72, 33%),* Enterococcus *species (18, 8%),* Streptococcus *species (17, 8%), and* Pseudomonas aeruginosa* (10, 5%).

Forty-two antibiotic-resistant bacteria strains were identified in 34 patients (Table 3), including 10* Pseudomonas aeruginosa* (24%), 8* Enterobacter *species (19%), seven ampicillin-resistant* Enterococcus faecium* (17%), 6 ESBL-producing* Klebsiella *species (14%), and 5 ESBL-producing* Escherichia coli* (12%). Of these, 23 bacteria (55%) were categorized into the SPACE group.

### 3.3. Risk Factors for Antibiotic-Resistant Bacteria

Differences between patients with and without antibiotic-resistant bacteria are shown in Table 4. Univariate analysis demonstrated that the Pitt bacteremia score, malignancy, prior appendectomy, antibiotic use within the preceding three months, indwelling biliary devices, hospitalization within the preceding 30 days, and bilioenteric anastomosis or sphincterotomy were statistically significant.

Multivariate analysis revealed that prior appendectomy (OR, 3.02; CI, 1.15–7.88; *p* = 0.026), antibiotic use within the preceding three months (OR, 3.06; 95% CI, 1.26–7.64; *p* = 0.013), and bilioenteric anastomosis or sphincterotomy (OR, 3.77; CI, 1.51–9.66; *p* = 0.0046) were the independent risk factors for antibiotic-resistant bacteria (Table 5). Of the 34 patients with antibiotic-resistant bacteremia, those with prior appendectomy were significantly younger than those without (66.4 ± 2.8 years old versus 73.9 ± 2.1 years old, *p* = 0.039), suggesting that appendectomy contributes to the development of antibiotic-resistant bacteria in relatively younger subjects.

## 4. Discussion

The present study demonstrates that history of appendectomy is a significant risk factor for development of antibiotic-resistant pathogens in bacteremia patients resulting from BTIs. Of the 174 patients examined, 34 developed bacteremia with antibiotic-resistant pathogens, 12 (35%) of which had a history of appendectomy, whereas only 20 (14%) of the remaining 140 subjects had the history. Because the lifetime risk of appendectomy has been reported to be 17.6% in the national hospital discharge survey [16], our cohort appeared to be common and hence the results could be generalized. In addition to prior appendectomy, antibiotic use within the preceding three months and bilioenteric anastomosis or sphincterotomy were found to be independent risk factors. These findings are consistent with those of previous studies [2, 4, 5] and also suggest generalizability of our study.

The immunological functions of the appendix may work for prevention of prevalence of antibiotic-resistant bacteria. The human appendix is associated with the highest concentration of gut-associated lymphoid tissue (GALT) in the gut [17]. GALT is a major source of plasma cell precursors, which migrate specifically to the intestinal lamina propria, and it is the site of initial CD4^+^ T cells activation [18]. Moreover, GALT are responsible for the generation of IgA-secreting cells and decreased colonic IgA (+) cells correlate with altered fecal microbiota composition [19]. In this regard, it has been noted that appendectomy markedly reduces the total intestinal plasma cells and immunoglobulins in the animal model [20]. In addition, the appendix is regarded as a natural immunologic barrier to bacterial translocation from the colon to the small bowel [10]. Thus, loss of the appendix by appendectomy may impair intestinal immunity and increase the risk of development of antibiotic-resistant bacteria of which virulence is usually attenuated.

Other functions of the appendix associated with intestinal flora may also be correlated with the results of the current study. The human appendix contains a wealth of microbes, including members of 15 phyla, which constitute more than 98% of the normal colonic microbiome [21]. This indicates that the appendix possesses a microbial diversity sufficient to reconstitute the microbiome of the intestine. To reinforce this concept, biofilms were found to be most abundant in the appendix [22]. Since biofilms are protective of bacteria, fragments of biofilms routinely shed from the appendix could serve as seeds for inoculation of the intestine with a normal microbial flora [23]. In this sense, the appendix, a potential harbor of normal flora for the intestines, can aid in the repopulation of commensal bacteria and lessen the uncontrollable overgrowth of abnormal, including antibiotic-resistant, bacteria in the intestine [24, 25]. This explanation matches the fact that the location of the appendix is secluded from the fecal stream, which presumably affords some protection from pathogenic organisms in gastrointestinal tract.

The present study indicates that appendectomy contributes to the development of antibiotic-resistant bacteria in relatively younger subjects with BTIs. Older subjects generally have more comorbid diseases and are relatively immunocompromised. Therefore, appendectomy may no longer affect their immunological defense. Alternatively, the functions of the appendix may attenuate in older subjects because the number of lymphoid follicles and length and diameter of the appendix are known to degenerate with age [26]. In clinical practice of bacteremia from BTI, a history of appendectomy should be noted, particularly in younger subjects.

Considering these facts, the appendix should no longer be regarded as a rudimentary organ and we should carefully decide whether appendectomy should always be indicated for appendicitis. Efficacy of conservative treatment with antibiotics on appendicitis is still controversial. A meta-analysis of randomized controlled trials showed that antibiotics were both effective and safe as primary treatment for patients with uncomplicated acute appendicitis [27]. The largest randomized clinical trial published in 2015 concluded that, among patients with uncomplicated appendicitis, antibiotic treatment did not meet the noninferiority compared with appendectomy [28], although over 70% of patients of the antibiotics group did not show recurrence within one year. The use of appendectomy or not for appendicitis should be determined by longer follow-up studies. Our results may be considered when making the choice of first-line therapy.

Based on the results of the current study, the recommended first choice of antibiotics for patients with bacteremia from BTI could be based on the presence or absence of identified risk factors including a history of appendectomy. In the Tokyo Guidelines [2], broad-spectrum antibiotics such as tazobactam/piperacillin, cefepime, cefozopran, ceftazidime, aztreonam ± metronidazole, and carbapenem are recommended for severe BTI and healthcare-associated BTI, whereas sulbactam/ampicillin, sulbactam/cefoperazone, cefazolin ± metronidazole, cefmetazole, and new quinolone ± metronidazole are indicated for others. For patients with the risks identified in this study, however, carbapenem plus vancomycin may be the first choice because the combination covers* Pseudomonas aeruginosa*, ampicillin-resistant* Enterococcus*, and ESBL-producing pathogens which were most frequently identified antibiotic-resistant pathogens in this study.

We demonstrated that history of appendectomy is a risk of development of antibiotic-resistant bacteria in patients with bacteremia from BTI. The majority of BTI patients, however, showed negative results in blood culture. The causative pathogens in these blood culture-negative BTI patients may also be different according to the risk factors including history of appendectomy. Although it may be difficult to verify the hypothesis due to difficulty in bacteria detection, bile culture could be an alternative, particularly in cases of cholangitis. Moreover, it is potentially useful to investigate whether causative microorganisms in a variety of infectious diseases other than BTI differ between patients with and without a history of appendectomy. Further studies are expected for these clinically relevant issues.

There are limitations to this study. First, the number of the examined patients was relatively small and all subjects were Japanese. Reproducibility should be confirmed with larger cohorts including other ethnic populations. Another drawback, due to the study being retrospective, is the possibility of missing relevant clinical parameters and inclusion of bias caused by missed blood culture at appropriate timing.

In conclusion, prior appendectomy was identified as an independent risk factor for antibiotic-resistant bacteria in BTIs. Before the choice of antibiotics, interviews regarding history of appendectomy are mandatory as well as other risk factors, including antibiotic use within the preceding three months and bilioenteric anastomosis or sphincterotomy.

## Figures and Tables

**Table 1:** Characteristics of patients with bacteremia from biliary tract infection.

Patients	
Total	174
Age (mean)	70.2 ± 13.9
Male	106 (61%)
Pitt bacteremia score (mean)	1.22 ± 1.21
Site of biliary tract infection	
Cholangitis	149 (86%)
Cholecystitis	25 (14%)
Cause of biliary tract infection	
No malignancy	116 (67%)
Malignancy	58 (33%)
Comorbidity	
Chronic kidney disease	47 (27%)
Diabetes mellitus	42 (24%)
Liver cirrhosis	21 (12%)
Medication	
Proton pump inhibitor	43 (25%)
Corticosteroids	22 (13%)
Prior appendectomy	32 (18.4%)
Antibiotic use within the preceding 3 months	57 (33%)
Indwelling biliary device	56 (32%)
Hospitalization within the preceding 30 days	51 (29%)
Bilioenteric anastomosis or sphincterotomy	42 (24%)
30-Day mortality	14 (8%)

**Table 2:** Spectrum of bacteria isolated from blood culture in patients with biliary tract infection.

Bacteria	Total (*N* = 221)
Gram-negative organisms	
*Klebsiella* species	83 (38%)
*Escherichia coli*	72 (33%)
*Pseudomonas aeruginosa*	10 (5%)
*Enterobacter* species	8 (4%)
*Proteus* species	3 (1%)
*Citrobacter* species	3 (1%)
*Serratia* species	1 (1%)
*Acinetobacter* species	1 (1%)
Gram-positive organisms	
*Enterococcus* species	18 (8%)
*Streptococcus* species	17 (8%)
*Staphylococcus* species	2 (1%)
Anaerobes	
*Bacteroides* species	3 (1%)

**Table 3:** Identified antibiotic-resistant bacteria.

Antibiotic-resistant bacteria	Total (*N* = 42)
SPACE	
*Pseudomonas aeruginosa*	10 (24%)
*Enterobacter* species	8 (19%)
*Citrobacter* species	3 (7%)
*Serratia* species	1 (2%)
*Acinetobacter* species	1 (2%)
Ampicillin-resistant *Enterococcus*	
*Enterococcus faecium*	7 (17%)
*Enterococcus faecalis*	1 (2%)
ESBL-producing *Klebsiella* species	6 (14%)
ESBL-producing *Escherichia coli*	5 (12%)

SPACE consists of *Serratia*, *Pseudomonas*, *Acinetobacter*, *Citrobacter*, and *Enterobacter*. ESBL, extended-spectrum *β*-lactamase.

**Table 4:** Differences between patients with and without antibiotic-resistant bacteria.

	Antibiotic-resistant bacteria		
	(+) (*N* = 34)	(−) (*N* = 140)	*p* value
Age (mean)	71	69	0.67
Male	19 (56%)	87 (62%)	0.50
Pitt bacteremia score (mean)	1.65	1.11	0.021
Site of biliary tract infection			
Cholangitis	29 (85%)	120 (86%)	0.95
Cholecystitis	5 (15%)	20 (14%)	0.95
Cause of biliary tract infection			0.058
No malignancy	18 (53%)	98 (70%)	
Malignancy	16 (47%)	42 (30%)	
Comorbidity			
Chronic kidney disease	10 (29%)	37 (26%)	0.73
Diabetes mellitus	8 (24%)	34 (24%)	0.92
Liver cirrhosis	2 (6%)	19 (14%)	0.21
Medication			
Proton pump inhibitor	9 (26%)	34 (24%)	0.80
Corticosteroids	5 (15%)	17 (12%)	0.69
Prior appendectomy	12 (35%)	20 (14%)	0.0046
Antibiotic use within the preceding 3 months	19 (56%)	38 (27%)	0.0014
Indwelling biliary device	15 (44%)	41 (29%)	0.090
Hospitalization within the preceding 30 days	15 (44%)	36 (26%)	0.035
Bilioenteric anastomosis or sphincterotomy	14 (41%)	28 (20%)	0.0096
30-Day mortality	4 (12%)	10 (7%)	0.79

**Table 5:** Risk factors for antibiotic-resistant bacteria (multivariate analysis).

	Odds ratio	*p*
	(95% confidence interval)	
Appendectomy	3.02 (1.15–7.87)	0.026^*∗*^
Pitt bacteremia score (per 1)	1.31 (0.54–1.48)	0.13
Malignancy	1.47 (0.60–3.56)	0.40
Antibiotic use within the preceding 3 months	3.06 (1.26–7.64)	0.013^*∗*^
Indwelling biliary device	1.34 (0.55–3.19)	0.51
Hospitalization within the preceding 30 days	2.45 (0.99–6.09)	0.051
Bilioenteric anastomosis or sphincterotomy	3.77 (1.51–9.66)	0.0046^*∗*^

*∗* means statistically significant.

## Abbreviations

BTI: Biliary tract infection
ESBL: Extended-spectrum *β*-lactamase
GALT: Gut-associated lymphoid tissue
SPACE: * Serratia*,* Pseudomonas*,* Acinetobacter*,* Citrobacter*, and* Enterobacter*.
